# Preemptive interferon-α therapy could prevent relapse of acute myeloid leukemia following allogeneic hematopoietic stem cell transplantation: A real-world analysis

**DOI:** 10.3389/fimmu.2023.1091014

**Published:** 2023-02-02

**Authors:** Shuang Fan, Tian-Zhong Pan, Li-Ping Dou, Yan-Min Zhao, Xiao-Hui Zhang, Lan-Ping Xu, Yu Wang, Xiao-Jun Huang, Xiao-Dong Mo

**Affiliations:** ^1^ Beijing Key Laboratory of Hematopoietic Stem Cell Transplantation, National Clinical Research Center for Hematologic Disease, Peking University People’s Hospital, Peking University Institute of Hematology, Beijing, China; ^2^ The First Affiliated Hospital of University of Science and Technology of China (USTC), Division of Life Sciences and Medicine, University of Science and Technology of China, Hefei, Anhui, China; ^3^ Department of Hematology, The First Medical Center of People's Liberation Army of China (PLA) General Hospital, Beijing, China; ^4^ Bone Marrow Transplantation Center, The First Affiliated Hospital, Zhejiang University School of Medicine, Hangzhou, China; ^5^ Peking-Tsinghua Center for Life Sciences, Academy for Advanced Interdisciplinary Studies, Peking University, Beijing, China; ^6^ Research Unit of Key Technique for Diagnosis and Treatments of Hematologic Malignancies, Chinese Academy of Medical Sciences, Beijing, China

**Keywords:** interferon-α, acute myeloid leukemia, hematopoietic stem cell transplantation, measurable residual disease, preemptive

## Abstract

**Introduction:**

Measurable residual disease (MRD)-directed interferon-a treatment (i.e. preemptive IFN-α treatment) can eliminate the MRD in patients with acute myeloid leukemia (AML) after allogeneic hematopoietic stem cell transplantation (allo-HSCT). Therefore, this study aimed to further assess its efficacy in a multicenter retrospective study in a real-world setting.

**Methods:**

A total of 247 patientswho received preemptive IFN-α treatment were recruited from 4 hospitals in China. The protocols for MRD monitoring mainly based on quantitative polymerase chain reaction [qPCR] and multiparameter flow cytometry [MFC].

**Results:**

The median duration of IFN-α treatment was 56 days (range, 1–1211 days). The cumulative incidences of all grades acute graft-versus-host disease (aGVHD), all grades chronic graft-versus-host disease (cGVHD), and severe cGVHD at 3 years after IFN-α therapy were 2.0% (95% confidence interval [CI], 0.3–3.8%), 53.2% (95% CI, 46.8–59.7%), and 6.2% (95% CI, 3.1–9.2%), respectively. The cumulative incidence of achieving MRD negative state at 2 years after IFN-α treatment was 78.2% (95% CI, 72.6–83.7%). The 3-year cumulative incidences of relapse and non-relapse mortality following IFN-α therapy were 20.9% (95% CI, 15.5–26.3%) and 4.9% (95%CI, 2.0–7.7%), respectively. The probabilities of leukemia-free survival and overall survival at 3 years following IFN-α therapy were 76.9% (95% CI, 71.5–82.7%) and 84.2% (95% CI, 78.7–90.1%), respectively. Multivariable analysis showed that MRD positive state by qPCR and MFC before IFN-α treatment, high-risk disease risk index before allo-HSCT, and receiving identical sibling donor HSCT were associated with a higher risk of relapse and a poorer leukemia-free survival. Severe cGVHD was associated with an increased risk of non-relapse mortality.

**Discussion:**

Thus, real-world data suggest that preemptive IFN-α is effective for treating patients with AML with MRD after allo-HSCT.

## Introduction

Allogeneic hematopoietic stem cell transplantation (allo-HSCT) is the most important curative therapy for acute myeloid leukemia (AML) and can significantly improve the survival of these patients (1, 2); however, relapse is still inevitable and is the most critical cause of treatment failure (3, 4). Measurable residual disease (MRD; previously termed minimal residual disease) can predict forthcoming relapse (5–9), and MRD-directed treatment (i.e., preemptive treatment) is the most important method to prevent relapse after allo-HSCT (7, 8). In addition, preemptive treatments allow patients with deep remission to avoid additional treatment.

Several methods have been proposed (e.g., donor lymphocyte infusion [DLI] (10–12), cytokine, and hypomethylating agents [HMAs] (13–15)) to be used for preemptive therapies. The immune effects of interferon-α (IFN-α) on AML cells (16, 17) rekindle interest in its utility after allo-HSCT (18–22). In addition, IFN-α therapy can be conveniently performed on an outpatient basis. For allo-HSCT recipients, the safety of IFN-α treatment has been confirmed (21, 23–25), and it could also clear the MRD effectively (26, 27). We observed that patients with AML who received preemptive IFN-α treatment could achieved persistent MRD negative state and long-term leukemia-free survival (LFS) in our single-center extension study (28).

However, these results were obtained from single center studies and have not been confirmed in multicenter studies. The samples in the above-mentioned studies were relatively small, and the efficacy of preemptive IFN-α treatment in some subgroups (e.g., patients with a high-risk disease risk index [DRI] before HSCT) should be further identified. Secondly, the previous studies did not compare the clinical outcome between children and adults, and whether children could achieve similar efficacy compared with adults after IFN-α treatment was unknown. Lastly, some authors observed that cGVHD after IFN-α was associated with a lower risk of relapse (24); however, other study did not report the association between cGVHD and relapse (27). In addition, severe cGVHD was associated with a higher risk of NRM after allo-HSCT (29). Thus, whether cGVHD could help to decrease the relapse and improve survival after IFN-α treatment was controversial.

Although MRD is a common complication after allo-HSCT, particularly for those with high-risk characteristics (30), the number of patients is limited, which prevents larger clinical studies from being appropriately conducted. Thus, real-world multicenter studies are similar to clinical practice and suitable for identifying the efficacy of preemptive IFN-α treatment.

Therefore, we conducted a multicenter study to further identify the efficacy of preemptive IFN-α treatment after allo-HSCT in the real-world setting. Furthermore, we also aimed to compare the efficacy of preemptive IFN-α treatment between adults and children.

## Methods

### Study design

**Figure 1:**
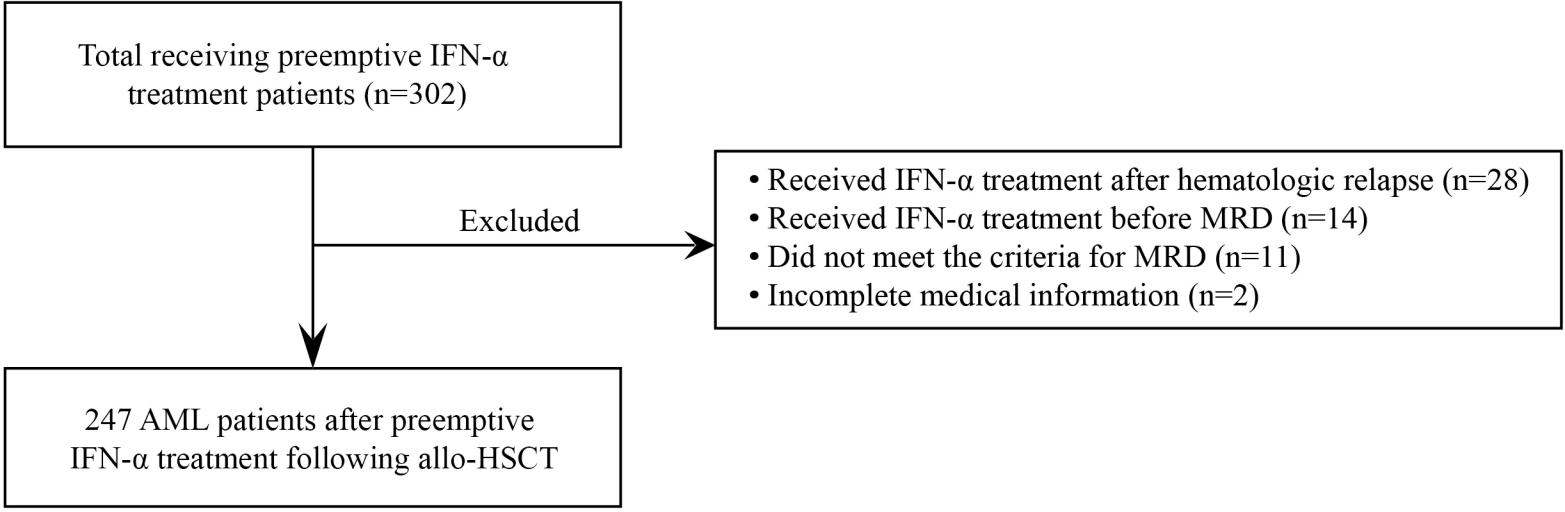
Diagram of enrolled patients.

We conducted a multicenter, retrospective study of patients with AML receiving preemptive IFN-α treatment across four hospitals in China. The protocols for MRD monitoring (based on quantitative polymerase chain reaction [qPCR] and multiparameter flow cytometry [MFC]) and the criteria for determining MRD positive state were mainly based on expert consensus on MRD monitoring in China (**Supplementary Methods**) (8, 31, 32). Patients eligible for the final analysis were allo-HSCT recipients (regardless of age, disease risk, and donor) with MRD who received at least one dose of IFN-α treatment. The exclusion criteria were as follows:1) patients who did not meet the criteria for MRD; 2) patients who received IFN-α treatment before MRD positive state; 3) patients who received IFN-α treatment after hematologic relapse; and 4) incomplete medical information (**Figure 1**); and 5) patients who received DLI or other preemptive treatment except IFN-α treatment. Patients were enrolled between January 1, 2017 and August 31, 2021. The final follow-up visit was on October 1, 2022. The study was approved by the Institutional Review Board of each hospital (2022PHB113-001) and was conducted in accordance with the *Declaration of Helsinki*.

### Data collection

The investigators at each hospital utilized a chart review and electronic medical records to obtain the required information, including patient demographics, diagnosis, DRI before HSCT, transplant regimen (e.g., donor type and donor-recipient relationship), MRD status after HSCT, preemptive IFN-α treatment (e.g., response after treatment), and clinical outcomes (e.g., relapse, mortality, and survival).

Two physicians independently reviewed the data to ensure the accuracy of the results.

### Transplant regimens

The protocol for preconditioning at each center was mainly based on the consensus from the Chinese Society of Hematology (33), and was also reported previously (**Supplementary Methods**) (4, 28, 33–42).

### Protocols of preemptive IFN-α therapy

Detailed information of IFN-α therapy is summarized in **Supplementary Methods**. Recombinant human IFN-α-2b can be initiated when MRD turns positive state, which also according to the intentions of patients and physicians, and based on the competence and experience of each center. The time interval between MRD positive state and IFN-α treatment is shown in **Table 1**. IFN-α injections were administered subcutaneously twice or thrice a week. For patients older than 16 years, IFN-α injections were administered at dosages of 3 million units; for those younger than 16 years, they were administered at 3 million units IU/m (2) (capped by 3 million units). Patients should also undergo MRD monitoring after preemptive IFN-α treatment. Immunosuppression and tapering strategies are shown in **Supplementary Methods** (28).

**Table 1:** Patient characteristics of patients receiving preemptive IFN-α therapy.

Characteristics	*n*=247
Sex, male/female, *n*	142/105
Median age at allo-HSCT, years (range)	34 (5–63)
First CR induction courses, *n* (%)	
1	176 (71.3)
> 1	71 (28.7)
Median duration from allo-HSCT to IFN-α therapy, days (range)	170 (31–1793)
Disease status at allo-HSCT, *n* (%)	
CR1	196 (79.4)
CR2	36 (14.6)
> CR2	15 (6.0)
FLT3 mutations at diagnosis, *n* (%)	
Yes	17 (6.9)
No	231 (93.1)
Disease risk index before allo-HSCT, *n* (%)	
Low-risk	53 (21.5)
Intermediate-risk	161 (65.2)
High-risk	33 (13.3)
MRD status before allo-HSCT, *n* (%)	
qPCR positive alone	146 (69.2)
MFC positive alone	12 (5.7)
qPCR and MFC positive at the same time	53 (25.1)
Donor–recipient relationship, *n* (%)	
Others	229 (92.7)
Mother–child	13 (5.2)
Donor-recipient sex matched, *n* (%)	
Others	209 (84.6)
Female to male	38 (15.4)
Donor type	
Identical sibling donor	64 (25.9)
Haploidentical donor	166 (67.3)
Unrelated donor	8 (3.2)
Unrelated cord blood	9 (3.6)
Number of HLA disparity (HLA-A, HLA-B, HLA-DR), *n* (%)	
0-1	80 (32.4)
2-3	167 (67.6)
Median duration from allo-HSCT to MRD positive state, days (range)	140 (25–1601)
Time from allo-HSCT to MRD positive state, *n* (%)	
Late-onset MRD	170 (68.8)
Early-onset MRD	77 (31.2)
MRD status before IFN-α therapy, *n* (%)	
qPCR positive alone	190 (76.9)
RUNX1-RUNX1T1	69 (27.9)
CBFβ-MYH11	17 (6.9)
WT1	76 (30.8)
Others	28 (11.4)
MFC positive alone	10 (4.0)
qPCR and MFC positive at the same time	47 (19.0)
Median duration from MRD to IFN-α therapy, days (range)	30 (0–276)
Median duration of follow-up after IFN-α therapy, days (range)	798 (24–2091)

IFN-α, interferon-α; allo-HSCT, allogeneic hematopoietic stem cell transplantation; MRD, measurable residual disease; CR, complete remission; HLA, human leukocyte antigen; qPCR, quantitative polymerase chain reaction; MFC, multiparameter flow cytometry.

### Definition and assessment

Disease status before allo-HSCT was evaluated using DRI (43). Graft-versus-host disease (GVHD) was diagnosed according to international criteria (44, 45). The definitions of late-onset MRD (LMRD), early-onset MRD (EMRD), relapse and, non-relapse mortality (NRM), LFS, and overall survival (OS) are shown in **Supplementary Methods** (26, 27).

### Statistical analysis

The primary endpoint was relapse, and the secondary endpoints included achieving MRD negative state, GVHD, NRM, LFS, and OS. To compare the characteristics of patients between groups, *χ* 2 and Fisher’s exact tests for categorical data and the Mann–Whitney *U*-test for continuous variable were performed. We used the Kaplan–Meier estimator to calculate the probabilities of OS and LFS. The cumulative incidence function was adopted to calculate the incidence of achieving MRD negative state, GVHD, relapse, and NRM with competing risk analysis (**Supplementary Methods**) (46). Univariable and multivariable Cox regression analyses are described in **Supplementary Methods**. Two-sided *P*-values were adopted. Statistical analysis was performed using the R software 4.2.0 (https://www.r-project.org) and Statistical Package for the Social Sciences 26 (SPSS Inc., IBM, Armonk, NY, USA).

## Results

### Patients characteristics

**Figure 2:**
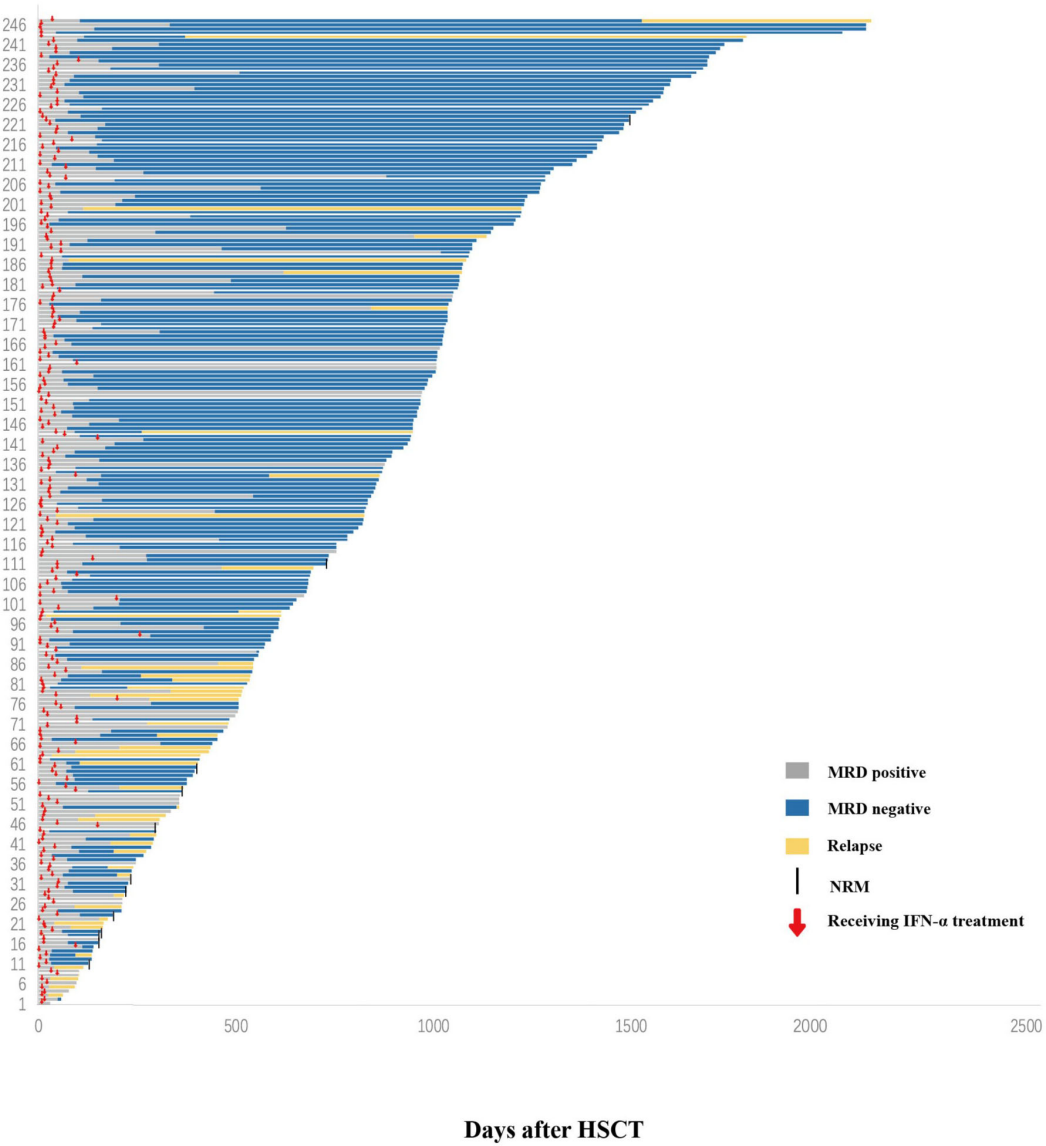
Response. Swimmer plot displaying all patients who received preemptive IFN-α therapy after allo-HSCT. IFN-α, interferon-α; allo-HSCT, allogeneic hematopoietic stem cell transplantation.

The characteristics of the 247 patients with AML receiving preemptive IFN-α therapy following allo-HSCT are summarized in **Table 1** and **Figure 2**, and information about human leukocyte antigen (HLA) disparity for haploidentical donor (HID) HSCT is shown in **Supplementary Table 1**. A total of 196 patients stopped calcineurin inhibitor (CNI) treatment before IFN-α treatment, and the other 51 with EMRD used IFN-α simultaneously with CNI, and CNI was gradually tapered and then ceased. The median duration from allo-HSCT to MRD positive state was 140 days (range, 25–1601) days. The median duration from allo-HSCT to IFN-α therapy was 170 days (range, 25–1877) days. The median duration from MRD positive state to IFN-α therapy was 30 days (range, 0–276) days. The median age of the patients receiving IFN-α was 34 years (range, 5–63) years, including 22 children (≤ 18 years) and 225 adults (> 18 years). The median duration of IFN-α therapy was 56 days (range: 1–1211 days). The median follow-up time was 798 days (range: 24–2091 days).

### GVHD

Five patients developed acute GVHD (aGVHD) after IFN-α treatment (**Supplementary Table 2**). The cumulative incidence of all grades aGVHD at 3 years after IFN-α therapy was 2.0% (95% CI, 0.3–3.8%). None of the patients experienced grades III–IV aGVHD, and 129 developed cGVHD after IFN-α treatment (**Supplementary Table 3**). The cumulative incidences of all grades and severe cGVHD at 3 years after IFN-α therapy were 53.2% (95% CI, 46.8–59.7%) and 6.2% (95% CI, 3.1–9.2%), respectively.

### Response after IFN-α treatment

A total of 190 (76.9%) patients achieved MRD negative state after IFN-α treatment, and 81 (42.6%), 33 (17.4%), 14 (7.4%), and 62 (32.6%) achieved MRD negative state 1, 2, 3, and > 3 months after preemptive IFN-α treatment, respectively. The median duration from IFN-α treatment to achieving MRD negative state results was 55 days (range: 7–948 days). The cumulative incidence of achieving MRD negative state at 2 years after IFN-α treatment was 78.2% (95% CI, 72.6–83.7%).

The cumulative incidence of achieving MRD negative state at 2 years after IFN-α treatment for adults and children was 79.7% (95% CI, 74.1–85.4%) and 58.8% (95% CI, 35.9–81.8%), respectively (*P =* 0.250). The cumulative incidence of achieving MRD negative state at 2 years after IFN-α treatment for patients receiving identical sibling donor (ISD), HID, and unrelated donor (URD)/unrelated cord blood (UCB) transplantation was 68.5% (95% CI, 56.5–80.4%), 82.8% (95% CI, 76.4–89.2%), and 67.9% (95% CI, 42.9–92.9%), respectively (*P =* 0.347, **Supplementary Figure 1A**). The cumulative incidence of achieving MRD negative state at 2 years after IFN-α treatment for patients in the low-, intermediate-, and high-risk DRI groups was 81.5% (95% CI, 69.3–93.7%), 81.0% (95% CI, 74.5–87.5%), and 57.6% (95% CI, 40.2–74.9%), respectively (*P =* 0.171, **Supplementary Figure 1B**). The cumulative incidence of achieving MRD negative state at 2 years after IFN-α treatment for patients with qPCR/MFC positivity alone and both qPCR and MFC positivity was 81.8% (95% CI, 75.9–87.6%) and 62.0% (95% CI, 47.0–76.9%), respectively (*P =* 0.035, **Supplementary Figure 1C**). The cumulative incidence of achieving MRD negative state at 2 years after IFN-α treatment for patients without chronic GVHD (cGVHD), with mild, moderate, and severe cGVHD was 77.4% (95% CI, 69.4–85.4%), 79.0% (95% CI 66.9–91.1%), 86.4% (95% CI, 75.7–97.2%), and 46.7% (95% CI, 20.1–73.3%), respectively (*P =* 0.149, **Supplementary Figure 1D**). The cumulative incidence of achieving MRD negative state at 2 years after IFN-α treatment was comparable among the adults and children without cGVHD, with mild, moderate, or severe cGVHD, respectively (**Supplementary Figure 2**).

### Relapse

Forty-eight patients experienced relapse following preemptive IFN-α therapy, and the median time from IFN-α therapy to relapse was 157 days (range: 3–1499 days). The cumulative incidence of relapse at 3 years after IFN-α therapy was 20.9% (95% CI, 15.5–26.3%).

**Figure 3:**
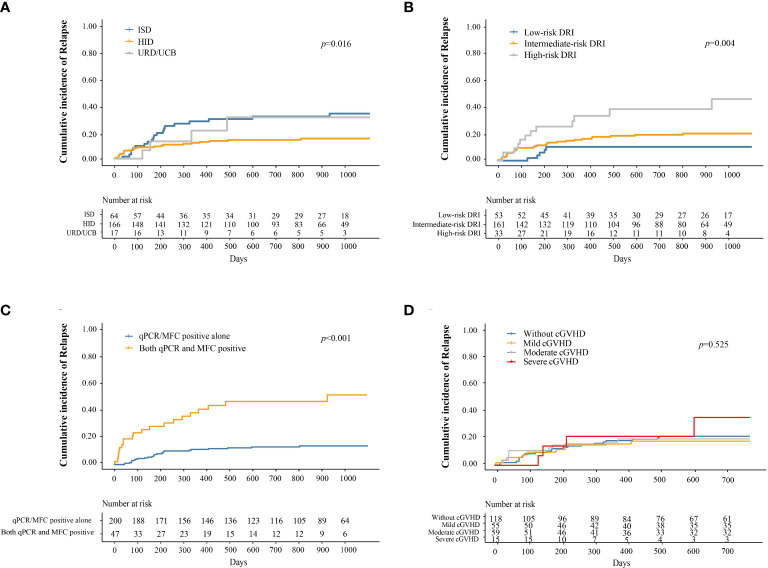
Cumulative incidence of relapse after preemptive IFN-α therapy for patients receiving ISD, HID, and URD/UCB transplantation **(A)**, for patients in the low-, intermediate-, and high-risk DRI groups **(B)**, for patients with qPCR/MFC positive alone and both qPCR and MFC positive **(C)**, and for patients without cGVHD, with mild, moderate, and severe cGVHD **(D)**. IFN-α, interferon-α; ISD, identical sibling donor; HID, haploidentical donor; URD, unrelated donor; UCB, unrelated cord blood; qPCR, quantitative polymerase chain reaction; MFC, multiparameter flow cytometry; DRI, disease risk index; cGVHD, chronic graft-versus-host disease.

The cumulative incidence of relapse at 3 years after IFN-α treatment in adults and children was 19.6% (95% CI, 14.2–25.1%) and 37.6% (95% CI, 8.0–67.2%), respectively (*P =* 0.273). The cumulative incidence of relapse at 3 years after IFN-α treatment for patients receiving ISD, HID, and URD/UCB transplantation was 33.5% (95% CI, 21.2–45.7%), 15.0% (95% CI, 9.2–20.7%), and 30.8% (95% CI, 3.3–58.2%), respectively (*P =* 0.016, **Figure 3A**). The cumulative incidence of relapse at 3 years after IFN-α treatment for patients in the low-, intermediate-, and high-risk DRI groups was 10.2% (95% CI, 1.6–18.7%), 20.0% (95% CI, 13.5–26.5%), and 45.3% (95% CI, 23.3–67.3%), respectively (*P =* 0.004, **Figure 3B**). The cumulative incidence of relapse at 3 years after IFN-α treatment for patients with qPCR/MFC positivity alone and both qPCR and MFC positivity was 14.0% (95% CI, 9.0–19.1%) and 52.3% (95% CI, 35.0–69.5%), respectively (*P <* 0.001, **Figure 3C**). The cumulative incidence of relapse at 2 years after IFN-α treatment for patients without cGVHD, with mild, moderate, and severe cGVHD was 19.9% (95% CI, 12.4–27.4%), 16.7% (95% CI, 6.6–26.8%), 18.2% (95% CI, 7.8–28.6%), and 33.1% (95% CI, 1.4–64.9%), respectively (*P =* 0.525, **Figure 3D**, the longest duration of LFS for patients with severe cGVHD was 952 days). In adults, the cumulative incidence of relapse at 2 years after IFN-α treatment was 18.0% (95% CI, 10.4–25.6%), 17.7% (95% CI, 7.1–28.4%), 17.7% (95% CI, 7.0%–28.4%) and 38.3% (95% CI, 3.4%–73.3%) in adults without cGVHD, with mild, moderate, or severe cGVHD (*P* = 0.059). The cumulative incidence of relapse at 2 years after IFN-α treatment was comparable among the children without cGVHD, with mild, moderate, or severe cGVHD (**Supplementary Figure 3**).

### NRM

Twelve patients experienced (infection, n = 10; GVHD, n = 2). The median duration from preemptive IFN-α therapy to NRM was 169 days (range 104–1474 days). The cumulative incidence of NRM at 3 years after IFN-α therapy was 4.9% (95% CI, 2.0–7.7%).

**Figure 4:**
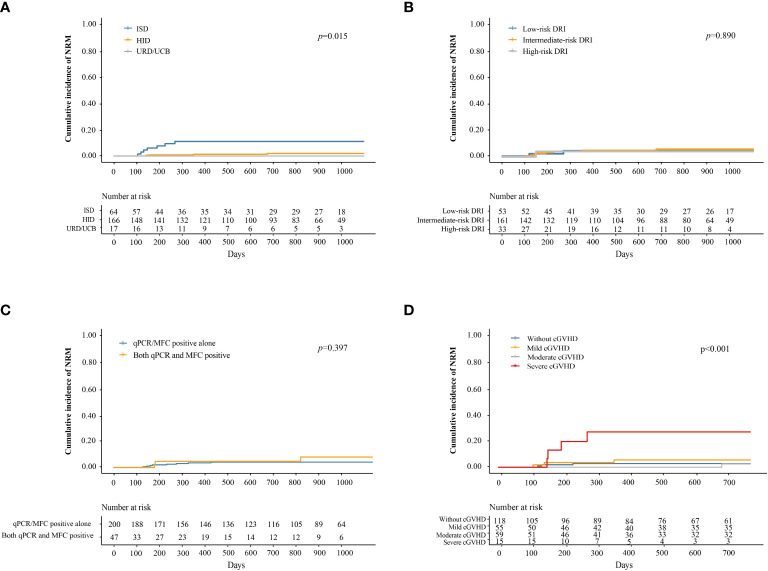
Cumulative incidence of NRM after preemptive IFN-α therapy for patients receiving ISD, HID, and URD/UCB transplantation **(A)**, for patients in the low-, intermediate-, and high-risk DRI groups **(B)**, for patients with qPCR/MFC positive alone and both qPCR and MFC positive **(C)**, and for patients without cGVHD, with mild, moderate, and severe cGVHD **(D)**. NRM, non-relapse mortality; IFN-α, interferon-α; ISD, identical sibling donor; HID, haploidentical donor; URD, unrelated donor; UCB, unrelated cord blood; qPCR, quantitative polymerase chain reaction; MFC, multiparameter flow cytometry; DRI, disease risk index; cGVHD, chronic graft-versus-host disease.

The cumulative incidence of NRM at 3 years after IFN-α treatment in adults and children was 5.3% (95% CI, 2.2–8.4%) and 0%, respectively (*P =* 0.296). The cumulative incidence of NRM at 3 years after IFN-α treatment in patients receiving ISD, HID, and URD/UCB transplantation was 11.5% (95% CI, 3.4–19.6%), 2.8% (95% CI, 0–5.6%), and 0%, respectively (*P =* 0.015, **Figure 4A**). The cumulative incidence of NRM at 3 years after IFN-α treatment for patients in the low-, intermediate-, and high-risk DRI groups was 4.0% (95% CI, 0–9.5%), 5.4% (95% CI, 1.7–9.1%), and 3.3% (95% CI, 0–9.7%), respectively (*P =* 0.890, **Figure 4B**). The cumulative incidence of NRM at 3 years after IFN-α treatment for patients with qPCR/MFC positivity alone and both qPCR and MFC positivity was 4.2% (95% CI, 1.3–7.0%) and 8.3% (95% CI, 0.0–17.7%), respectively (*P =* 0.397, **Figure 4C**). The cumulative incidence of NRM at 2 years after IFN-α treatment in patients without cGVHD, with mild, moderate, and severe cGVHD was 2.7% (95% CI, 0.0–5.6%), 5.6% (95% CI, 0–11.9%), 2.6% (95% CI, 0–7.6%), and 27.5% (95% CI, 3.2–51.8%), respectively (*P <* 0.001, **Figure 4D**). In adults, the cumulative incidence of NRM at 2 years after IFN-α treatment was 3.0% (95% CI, 0–6.3%), 6.0% (95% CI, 0–12.6%), 2.7% (95% CI, 0%–8.1%) and 35.0% (95% CI, 4.8%–65.2%) in adults without cGVHD, with mild, moderate, or severe cGVHD (*P* < 0.001) (**Supplementary Figure 4**).

### LFS

**Figure 5:**
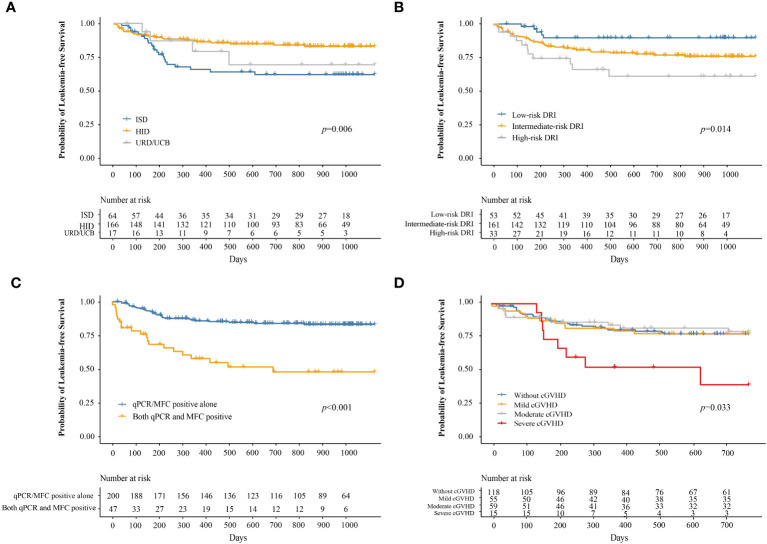
The probability of LFS after preemptive IFN-α therapy for patients receiving ISD, HID, and URD/UCB transplantation **(A)**, for patients in the low-, intermediate-, and high-risk DRI groups **(B)**, for patients with qPCR/MFC positive alone and both qPCR and MFC positive **(C)**, and for patients without cGVHD, with mild, moderate, and severe cGVHD **(D)**. LFS, leukemia-free survival; IFN-α, interferon-α; ISD, identical sibling donor; HID, haploidentical donor; URD, unrelated donor; UCB, unrelated cord blood; qPCR, quantitative polymerase chain reaction; MFC, multiparameter flow cytometry; DRI, disease risk index; cGVHD, chronic graft-versus-host disease.

At 3 years after IFN-α therapy, the probability of LFS was 76.9% (95% CI, 71.5–82.7%). The probabilities of LFS at 3 years after IFN-α treatment for adults and children were 77.1% (95% CI, 71.5–83.2%) and 74.9% (95% CI, 57.8–97.0%), respectively (*P =* 0.780). The probability of LFS at 3 years after IFN-α treatment for patients receiving ISD, HID, and URD/UCB transplantation was 61.9% (95% CI, 50.5–76.0%), 82.9% (95% CI, 77.0–89.2%), and 69.2% (95% CI, 47.6–100%), respectively (*P =* 0.006, **Figure 5A**). The probabilities of LFS at 3 years after IFN-α treatment for patients in the low-, intermediate-, and high-risk DRI groups were 89.7% (95% CI, 81.5–98.7%), 75.8% (95% CI, 69.1–83.1%), and 60.9% (95% CI, 44.8–82.8%), respectively (*P =* 0.014, **Figure 5B**). The probability of LFS at 3 years after IFN-α treatment for patients with qPCR/MFC positivity alone and both qPCR and MFC positivity was 83.2% (95% CI, 77.9–88.8%) and 47.9% (95% CI, 34.2–67.2%), respectively (*P <* 0.001, **Figure 5C**). The probability of LFS at 2 years after IFN-α treatment for patients without cGVHD, with mild, moderate, and severe cGVHD was 77.4% (95% CI, 70.0–85.6%), 77.6% (95% CI, 67.2–89.7%), 79.2% (95% CI, 68.8–91.2%), and 39.4% (95% CI, 18.6–83.2%), respectively (*P =* 0.033, **Figure 5D**). In adults, the probability of LFS at 2 years after IFN-α treatment was 79.0% (95% CI, 71.4–87.5%), 76.3% (95% CI, 65.4–89.0%), 80.0% (95% CI, 68.8%–92.0%) and 26.7% (95% CI, 9.1%–78.0%) in adults without cGVHD, with mild, moderate, or severe cGVHD (*P* = 0.001). The probability of LFS at 2 years after IFN-α treatment was comparable among the children without cGVHD, with mild, moderate, or severe cGVHD (**Supplementary Figure 5**).

### OS

**Figure 6:**
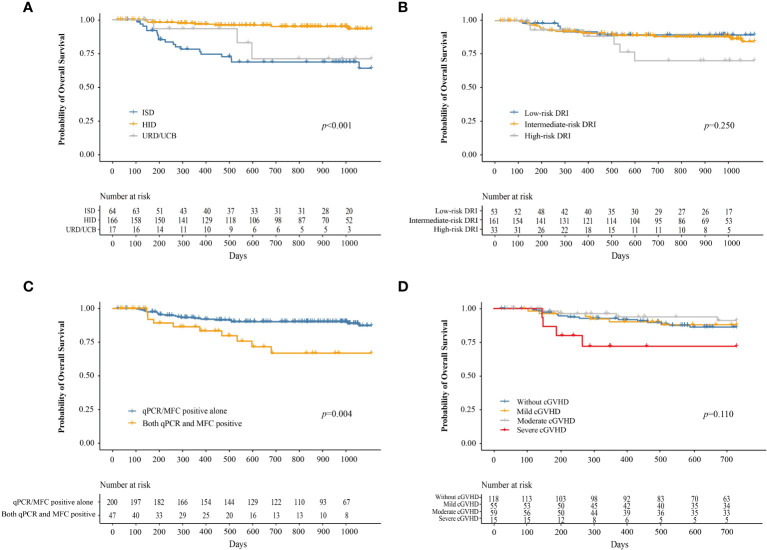
The probability of OS after preemptive IFN-α therapy for patients receiving ISD, HID, and URD/UCB transplantation **(A)**, for patients in the low-, intermediate-, and high-risk DRI groups **(B)**, for patients with qPCR/MFC positive alone and both qPCR and MFC positive **(C)**, and for patients without cGVHD, with mild, moderate, and severe cGVHD **(D)**. OS, overall survival; IFN-α, interferon-α; ISD, identical sibling donor; HID, haploidentical donor; URD, unrelated donor; UCB, unrelated cord blood; qPCR, quantitative polymerase chain reaction; MFC, multiparameter flow cytometry; DRI, disease risk index; cGVHD, chronic graft-versus-host disease.

At 3 years after IFN-α therapy, the probability of OS was 84.2% (95% CI, 78.7–90.1%). The probability of OS at 3 years after IFN-α treatment for adults and children was 84.5% (95% CI, 78.8–90.6%) and 80.6% (95% CI, 62.7–100%), respectively (*P =* 0.730). The probability of OS at 3 years after IFN-α treatment for patients receiving ISD, HID, and URD/UCB transplantation was 64.1% (95% CI, 51.3–80.0%), 93.2% (95% CI, 88.4–98.3%), and 71.1% (95% CI, 47.5–100%), respectively (*P* < 0.001, **Figure 6A**). The probabilities of OS at 3 years after IFN-α treatment for patients in the low-, intermediate-, and high-risk DRI groups were 89.4% (95% CI, 81.0–98.7%), 84.5% (95% CI, 77.6–91.9%), and 70.3% (95% CI, 52.2–94.6%), respectively (*P =* 0.250, **Figure 6B**). The probability of OS at 3 years after IFN-α treatment for patients with qPCR/MFC positivity alone and both qPCR and MFC positivity was 87.2% (95% CI, 81.5–93.2%) and 66.7% (95% CI, 51.2–87.0%), respectively (*P =* 0.004, **Figure 6C**). The probability of OS at 2 years after IFN-α treatment for patients without cGVHD, with mild, moderate, and severe cGVHD was 86.2% (95% CI, 79.7–93.3%), 88.0% (95% CI, 79.3–97.5%), 91.2% (95% CI, 83.1–100%), and 72.0% (95% CI, 86.7–100%), respectively (*P =* 0.110, **Figure 6D**). In adults, the probability of OS at 2 years after IFN-α treatment was 88.3% (95% CI, 81.9–95.1%), 87.2% (95% CI, 78.0–97.4%), 90.7% (95% CI, 82.2%–100.0%) and 64.3% (95% CI, 41.2%–100.0%) in adults without cGVHD, with mild, moderate, or severe cGVHD (*P* = 0.025). The probability of OS at 2 years after IFN-α treatment was comparable among the children without cGVHD, with mild, moderate, or severe cGVHD (**Supplementary Figure 6**).

### Infection

A total of 69 (27.9%) patients experienced infection after preemptive IFN-α treatment. The most common site of infection was pulmonary infection (n = 39), followed by upper respiratory tract infection (n = 22), intestinal infection (n = 4) and other infection (n = 4). A total of 10 patients died of pulmonary infection.

### Multivariable analysis

Multivariable analysis showed that both qPCR and MFC positive before IFN-α treatment, high-risk disease risk index before allo-HSCT, and receiving identical sibling donor allo-HSCT were associated with a higher risk of relapse and poorer LFS. Severe cGVHD was associated with an increased risk of NRM. Both qPCR and MFC positive before IFN-α treatment and receiving allo-HSCT from an identical sibling donor were associated with a poorer OS (**Table 2** and **Supplementary Table 4**).

**Table 2:** Multivariate analysis of risk factors for 3-year clinical outcomes after preemptive IFN-α treatment.

Outcome	HR (95% CI)	*P*
Relapse		
Disease risk index before allo-HSCT		
Low risk	1	
Intermediate risk	2.21 (0.86–5.68)	0.101
High risk	4.03 (1.40–11.58)	0.010
MRD status before IFN-α therapy		
qPCR/MFC positive alone	1	
qPCR and MFC positive at the same time	4.04 (2.24–7.26)	<0.001
Donor type		
Identical sibling donor	1	
Alternative donor	0.55 (0.31–0.99)	0.046
NRM		
Severity of cGVHD after preemptive IFN-α therapy		
None	1	
Mild to moderate	1.59 (0.38–6.69)	0.524
Severe	9.98 (2.08–47.91)	0.004
Treatment failure as defined by LFS		
Disease risk index before allo-HSCT		
Low risk	1	
Intermediate risk	2.03 (0.91–4.53)	0.085
High risk	3.28 (1.29–8.32)	0.012
MRD status before IFN-α therapy		
qPCR/MFC positive alone	1	
qPCR and MFC positive at the same time	3.48 (2.04–5.91)	<0.001
Donor type		
Identical sibling donor	1	
Alternative donor	0.48 (0.29–0.80)	0.008
Treatment failure as defined by OS		
Donor type		
Identical sibling donor	1	
Alternative donor	0.26 (0.12–0.55)	<0.001
MRD status before IFN-α therapy		
qPCR/MFC positive alone	1	
qPCR and MFC positive at the same time	2.35 (1.12–4.94)	0.025

IFN-α, interferon-α; CI, confidence interval; HR, hazard ratio; allo-HSCT, allogeneic hematopoietic stem cell transplantation; CR, complete remission; MRD, measurable residual disease; qPCR, quantitative polymerase chain reaction; MFC, multiparameter flow cytometry; cGVHD, chronic graft-versus-host disease; NRM, non-relapse mortality; LFS, leukemia-free survival; OS, overall survival.

## Discussion

In this large-scale multicenter study, more than 75% of the patients achieved MRD negative state, and the probabilities of relapse, NRM, and LFS at 3 years after preemptive IFN-α therapy were 20.9%, 4.9%, and 76.9%, respectively. These results were similar to our single center-study; the incidence of relapse, NRM, and LFS was 13.0%, 3.9%, and 83.1% after IFN-α treatment (28). To our knowledge, these findings are the first to confirm the clinical value of preemptive IFN-α treatment in AML patients following allo-HSCT in the real world.

We observed that patients with severe cGVHD after IFN-α treatment showed a lower rate of achieving MRD negative state and a higher incidence of relapse. This may be due to the intense and long-term immunosuppressive therapies for severe cGVHD that might abrogate the graft-versus-leukemia effect (47). In addition, we observed that severe cGVHD after IFN-α treatment was associated with a higher risk of NRM in multivariable analysis. Considering that severe cGVHD can cause mortality and morbidity and negatively influence health-related quality of life (48–51), it should be prevented after IFN-α treatment. However, only 6% of patients experienced severe cGVHD in this multicenter study, which suggests that the intensity of cGVHD induced by IFN-α treatment was under control.

In addition, the incidence of NRM at 3 years after IFN-α treatment was only 4.9%, which was in accordance with our single-center study (26–28), and was similar to patients with persistent MRD negative state after allo-HSCT (24). Thus, the safety of preemptive IFN-α treatment was further confirmed in a real-world study.

We observed that the incidence of response, relapse, and LFS after IFN-α was 67.9%, 30.8% and 69.2% of patients receiving URD/UCB HSCT, respectively, which was comparable in patients receiving ISD or HID allo-HSCT. It is suggested that URD/UCB HSCT recipients could benefit from preemptive IFN-α treatment, which is important because these patients had difficulty receiving further preemptive cellular therapy (e.g., DLI) after allo-HSCT.

We observed that the clinical outcomes of patients in the high-risk DRI group were poor; only half of them could achieve MRD negative state, and the incidence of relapse was as high as 45.3%. Several studies have reported that DRI could predict clinical outcomes after allo-HSCT (52–54), and we firstly observed that it could also predict outcomes after preemptive IFN-α treatment following allo-HSCT.

Other methods can be used to prevent or treat relapse after allo-HSCT. Several studies have used HMAs as a preemptive treatment in patients with AML following allo-HSCT. However, the long-term efficacy of HMAs treatment seems unsatisfactory despite the delay in hematologic relapse (13–15). Venetoclax-based regimens are an important therapy for patients with refractory/relapsed AML (55, 56). Fang et al. (57) using venetoclax-based regimens for patients with AML with MRD. They observed that the major response rate was 50%, and the relapse-free survival of responsive patients was significantly prolonged. Thus, the efficacy of these methods is worthy of identification in patients with high-risk DRI (58).

We observed that patients with both qPCR and MFC positivity had a higher risk of relapse and a poorer LFS after IFN-α treatment. In a study by Zhao et al. (59), the relapse rate of patients with AML who had both qPCR and MFC positivity was as high as 92.3%. These patients may benefit more from DLI because their efficacy was confirmed by Yan et al. (10).

There were 21.5% of patients categorized into the low-risk DRI group before HSCT, and most t(8;21) AML. Zhu et al. (60) reported that patients who could not achieve major molecular remission (MMR, i.e., achieved a ≥ 3-log reduction after the second consolidation and/or the loss of a ≥ 3-log reduction during the next six consolidation therapies) were at high risk for relapse, and allo-HSCT could significantly improve their outcomes. Thus, patients with t(8;21) AML who cannot achieve MMR are also recommended to receive allo-HSCT as consolidation treatment in the consensus from the Chinese Society of Hematology (33).

Because of the shortage of donors with ISDs and URDs in China, HIDs HSCT is a valuable option in transplant procedures. HIDs have accounted for 60% of all of the allo-HSCT in China (42). This may contribute to the fact that our cohort has a high number of HID HSCT recipients.

In our previous studies, most of the patients were adults (7, 26), and the efficacy of preemptive IFN-α treatment in children was unclear. In the present study, we observed that the 2-year incidence of achieving MRD negative state was 58.8%, and the 3-year probabilities of relapse, LFS and OS after IFN-α treatment were 37.6%, 74.9%, and 80.6% respectively, in children, which were comparable with those of adults. Thus, children could also benefit from preemptive IFN-α treatment. However, only 22 children were enrolled in this study. Therefore, it is still premature to conclude the comparable clinical outcomes between adults and children receiving preemptive IFN-α treatments, and a prospective study with a larger sample of children should further confirm these results.

This study had some limitations. Residual confounding was unavoidable; however, this is a common problem in retrospective studies based on electronic medical records. Only nine UCBT recipients were enrolled, and we could not further identify the efficacy of preemptive IFN-α treatment in these patients. Only a small number of patients experienced NRM (n = 12, 4.9%) in the present study, which may influence the further comparison of NRM occurrence among patients with different donors. In addition, only 17 patients received URD/UCBT HSCT, and no one happens to experience NRM in this small cohort. Although the incidence of NRM seemed to be higher in the ISD HSCT group than that in the HID HSCT group, the cause of NRM was comparable between the two groups (infection: 85.7% vs. 80.0%, *P* = 1.000; GVHD: 14.3% vs. 20.0%, *P* = 1.000). These results should be further confirmed in a prospective study with a larger sample of URD/UCBT HSCT recipients. In addition, this was a retrospective multicenter study, and MRD monitoring was performed in different laboratories. However, all participating hospitals are the largest and most experienced centers for allo-HSCT in China and have extensive experience in MRD monitoring. Prospective studies that perform MRD monitoring in a central laboratory may further help confirm the efficacy of preemptive IFN-α treatment. The starting point of the analysis was the first day of IFN-α treatment, and patients with acute relapse, refractory diseases, or early NRM were excluded from the analysis. However, if we used the first day of allo-HSCT as the starting point for analysis, the outcomes of patients who showed late-onset MRD (e.g., several years after allo-HSCT) could not reflect the real long-term efficacy of IFN-α treatment and may also introduce a false-positive effect on the results. Finally, this was a single-arm study, and future clinical trials are required to compare the efficacy of IFN-α treatment with other preemptive treatments (e.g., azacitidine).

Therefore, this large-scale real-world study supports the utility of IFN-α treatment for treating patients with AML with MRD after allo-HSCT. Future prospective randomized controlled trials are essential to compare the efficacy of IFN-α and other preemptive treatments.

## Data availability statement

The original contributions presented in the study are included in the article/**Supplementary Material**. Further inquiries can be directed to the corresponding author.

## Ethics statement

Ethical review and approval was not required for the study on human participants in accordance with the local legislation and institutional requirements. Written informed consent was obtained from the individual(s), and minor(s)’ legal guardian/next of kin, for the publication of any potentially identifiable images or data included in this article.

## Author contributions

X-DM and X-JH designed the study. SF, T-ZP, L-PD, Y-MZ, X-HZ, L-PX, and YW conducted data collection. SF, T-ZP and X-DM conducted data analysis and drafted manuscript. All authors contributed to the article and approved the submitted version.
