# Supplementary material for: Preemptive interferon-α therapy could prevent relapse of acute myeloid leukemia following allogeneic hematopoietic stem cell transplantation: A real-world analysis
Source: Front Immunol. 2023 Feb 2;14:1091014. doi: 10.3389/fimmu.2023.1091014 (PMC9932895; doi:10.3389/fimmu.2023.1091014)
Supplement: Supplementary file 1 [file DataSheet_1.docx]

**Supplemental Appendix**

**Preemptive interferon-α therapy could prevent relapse of acute myeloid leukemia following allogeneic hematopoietic stem cell transplantation: a real-world analysis.**

**Short title: IFN-α prevent relapse after allo-HSCT**

**Contents**

**Supplementary Methods** ............................................................................................ 2

Conditioning regimen..................................................................................................................2

Immunosuppression and tapering strategies..................................................................................2

MRD monitoring after Allo-HSCT.............................................................................................. 3

Preemptive IFN-α therapy protocol ............. ..................................................................................3

Definitions and assessments ......................................................................................................... 3

Statistical analysis ........................................................................................................................ 3

**Supplementary Tables** ..............................................................................................5

Supplementary Table 1. Information about HLA disparity .......... ......................................... 5

Supplementary Table 2. Characteristics of aGVHD after preemptive IFN-α therapy ............ 6

Supplementary Table 3. Characteristics of cGVHD after preemptive IFN-α therapy ............ 7

Supplementary Table 4. Univariable analysis of prognostic factors following preemptive IFN-α therapy ……………………………………………………………………………………………. 8

**Supplementary Figure** ............................................................................................ 11

Supplementary Figure 1............................ .................................................................................. 11

Supplementary Figure 2............................ .................................................................................. 12

Supplementary Figure 3............................ .................................................................................. 13

Supplementary Figure 4............................ .................................................................................. 14

Supplementary Figure 5............................ .................................................................................. 15

Supplementary Figure 6............................ .................................................................................. 16

**References**........................................................................................ ..........................17

**Supplementary methods**

**Conditioning regimen**

In Peking University People's Hospital, the principal myeloablative preconditioning regimen was cytosine arabinoside (Ara-C), busulfan (Bu, 3.2 mg/kg/day, day -8, day -7, and day -6), cyclophosphamide (Cy, 1.8 g/m^2^/day, day -5 and day -4), and simustine (250 mg/m^2^, day -3). Ara-C was administered at 4 g/m^2^/day (day -10 and day -9) to the HID group, at 2g/m^2^/day (day -10 and day -9) to the URD group, and at 2 g/m^2^/day (day -9) to the ISD group. In addition, HID and URD groups received rabbit anti-thymocyte globulin (thymoglobulin, 2.5mg/kg/d, day -5, day -4, day -3, and day -2; Sanofi, France) to prevent GVHD^1^.

In the First Affiliated Hospital of URD and UCB HSCT recipients received a myeloablative conditioning

regimen of BUCY2 (Bu [varying between 0.8 and 1.2 mg/kg based on the patient’s body weight every 6 h for 4 days] and CY (60 mg/kg daily for 2 days)] or TBI/CY (TBI, total 12 Gy, 4 fractions and CY 60 mg/kg daily for 2 days). Ara-c (2.0 g/m2 every 12 h for 2 days) and fludarabine (30 mg/m2 daily for 4 days) were combined with BUCY2 or TBI/CY to promote engraftment. In some patients, carmustine (250 mg/m2) was added to the myeloablative conditioning regimen to further kill the leukemia cells, especially in extramedullary sites^2^.

In the first medical center of PLA General Hospital, ISD and HID HSCT recipients received modified Bu/Cy conditioning regimen consisted of Bu (3.2 mg/kg/day, days –10 to −8), carmustine (250 mg, day −7), cytarabine (4 g/m2/day, days −6 to− 5), and Cy (50 mg/kg/d, days − 4 to − 3). The patients with organ dysfunction during previous chemotherapy received Flu/Bu conditioning regimen, which was similar to the modified Bu/Cy regimen except that Cy was substituted with fludarabine (Flu, 30 mg/m2/day, days −7 to −3). ATG (thymoglobulin, rabbit; Genzyme Europe BV; 2.5 mg/kg/day, days −5 to −2) was used in all the HID HSCT recipients^3^.

In the First Affiliated Hospital, Zhejiang University School of Medicine, patients receiving HID-HSCT or URD-HSCT, the main myeloablative conditioning regimen used involved Bu (3.2 mg/kg/d IV on days –7 to –4), Cy (60 mg/kg/d IV on days –3 to –2). For those patients with central nervous system leukemia or a high WBC count at diagnosis or failure to achieve CR before HSCT, the conditioning regimen consisted of cytarabine (2 g/m2 per day IV on days –8 to –7), Bu (3.2 mg/kg per day IV on days –6 to –4), Cy (1.8 g/m2 per day IV on days –3 to –2), and methyl-N-(2-chloroethyl)-N-cyclohexyl-N-nitrosourea (250 mg/m2 orally on day –1). Rabbit ATG (Thymoglobulin; Genzyme, Cambridge, MA) was also administered to patients receiving URD-HSCT (4.5-6 mg/kg total dose). For patients receiving HID-HSCT, the conditioning regimen consisted of cytarabine (4 g/m2/d IV on days –10 to –9), Bu (3.2 mg/kg per day IV on days –8 to –6), Cy (1.8 g/m2 per day IV on days –5 to –4), methyl-N-(2-chloroethyl)-N-cyclohexyl-N-nitrosourea (250 mg/m2 orally on day –3), and anti-T-lymphocyte globulin (ATG-F; Fresenius, Bad Homburg, Germany) (2.5 mg/kg per day IV on days –5 to –2) ^4^.

**Immunosuppression and tapering strategies**

All patients with ISD, HID or URD received calcineurin inhibitor (CNI), mycophenolate mofetil (MMF), and short-term MTX for graft-versus-host disease (GVHD) prophylaxis. UCBT recipients received CNI and MMF as GVHD prophylaxis.

CNI was tapered/stopped according to the time of MRD occurring. Patients with early-onset MRD (EMRD, i.e., < 100 days after allo-HSCT) could use IFN-α simultaneously with CNI, and CNI was gradually tapered and then ceased if the patients did not show new onset GVHD (n=51). These patients could also stop CNI before IFN-α treatment (n=26). For the patients with late-onset MRD (LMRD, i.e., ≥ 100 days after allo-HSCT) group, CNI should be stopped before they received IFN-α treatment (n=170) ^1^.

**MRD monitoring after Allo-HSCT**

Leukemia-associated aberrant immunophenotypes (LAIPs) is identified by MFC and 0.1% was used as a threshold to distinguish MRD-positive from MRD-negative patients with AML. Quantitative PCR assays for MRD monitoring are based on the detection of leukemic-specific targets, such as a fusion gene, mutated or overexpressed gene. It is recommended to regularly detect MRD status at +1, +2, +3, +4 (or +4.5), +6, +9, +12, +18, +24, +36, +48, and +60 months after transplantation. Each center can also follow its own schedules according to the actual situation, and increase the frequency of testing when necessary. Patients with detectable MRD could re-check within two weeks ^5^.

**Preemptive IFN-α therapy protocol**

Recombinant human IFN-α-2b injections (Anferon; Tianjin Hualida Biotechnology Co., Ltd., Tianjin, China) were administered subcutaneously for 6 cycles (twice weekly in every 4 weeks cycle) at dosages of 3 million units for patients older than 16 years, and at 3 million units per square meter for those younger than 16 years (capped by 3 million units). Prolonged treatment with IFN-α was permitted at the request of patients. MRD status was monitored 1, 2, 3, 4.5, 6, 9, and 12 months after preemptive IFN-α therapy and at 6-month intervals thereafter. Study medication with IFN-α was discontinued in any patient with active GVHD (grade II or higher aGVHD or cGVHD with moderate or higher severity), severe infection, grade ≥ 3 toxicity, salvage Chemo-DLI use, relapse, or non-relapse mortality (NRM).

**Definitions and assessments**

Relapse was defined as recurrence of BM blasts >5%, reappearance of blasts in the blood, development of extramedullary disease infiltrates at any site, or by the recurrence and sustained presence of pre-transplantation chromosomal abnormalities after IFN-α treatment. NRM was defined as death without disease progression or relapse after IFN-α treatment. Overall survival (OS) events were defined as death from any cause after IFN-α treatment. Leukemia-free survival (LFS) was defined as the survival period with continuous complete remission (CR) after IFN-α treatment. Early-onset MRD (EMRD) was defined as testing positive for MRD < 100 days after allo-HSCT, and late-onset MRD (LMRD) was defined as testing positive for MRD ≥ 100 days after allo-HSCT.

**Statistical analysis**

The cumulative incidence function was adopted to calculate the incidence of MRD achieving negativity, GVHD, relapse, and NRM with competing risk analysis. For MRD achieving negativity and GVHD, relapse and NRM were competing risks. For NRM, relapse was competing risks. For relapse, NRM was competing risks. Univariable and multivariable Cox regression analysis were utilized to estimate hazard ratios (HRs) for clinical outcomes. The Cox proportional hazard model we established included variables as follows: patient gender, age (≤18 *vs*. >18 years), first CR induction courses (1 *vs.* >1), disease risk index before allo-HSCT (low risk *vs.* intermediate risk *vs.* high risk), MRD status before allo-HSCT (negative *vs.* positive), donor type (matched sibling donor *vs.* alternative donor), donor/recipient gender matched (female donor/male recipient combination *vs.* others), MRD status before IFN-α therapy (qPCR/MFC positive alone *vs.* qPCR and MFC positive at the same time), time from allo-HSCT to MRD positive (LMRD *vs.* EMRD), and severity of cGVHD after preemptive IFN-α therapy (none *vs.* mild to moderate *vs.* severe cGVHD). The factors associated with the clinical outcomes with *P* < 0.1 by univariable analysis were included in the multivariable analysis using the Cox proportional-hazard regression model. The cutoff significance level of 0.05 was adopted in the stepwise backward procedure for removal of variables from the model.

**Supplementary Tables**

**Supplementary Table 1.** **Detailed information about HLA disparity**

| Location of HLA antigen mismatched | N (%) |
| --- | --- |
| Only HLA-A | 4 (2.3) |
| Only HLA-B | 2 (1.1) |
| Only HLA-DR | 2 (1.1) |
| Mismatches at HLA-A and -B | 5 (2.9) |
| Mismatches at HLA-A and -DR | 7 (4.0) |
| Mismatches at HLA-B and -DR | 15 (8.6) |
| Mismatches at HLA-A,-B, and -DR | 140 (80.0) |
| Total | 175 (100) |

HLA, human leukocyte antigen

**Supplementary Table 2. Characteristics of aGVHD after preemptive IFN-α treatment**

| **Characteristics of aGVHD** | ***n* =247** |
| --- | --- |
| Median duration from IFN-α treatment | 4 (0–19) |
| Severity of aGVHD, *n* (%) |  |
| None | 242 (97.5) |
| Grade I | 2 (0.81) |
| Grade II | 3 (1.21) |
| Grade III | 0 (0.0) |
| Grade IV | 0 (0.0) |
| Site of aGVHD, *n* (%) |  |
| Skin | 4 (1.6) |
| Gut | 1 (0.9) |
| Number of sites, *n* (%) |  |
| 0 | 242 (97.5) |
| 1 | 5 (2.5) |
| 2 | 0 (0.0) |
| 3 | 0 (0.0) |

aGVHD, acute graft-versus-host disease; IFN-α, interferon-α

**Supplementary Table 3. Characteristics of cGVHD after preemptive IFN-α treatment**

| **Characteristics** | ***n* =247** |
| --- | --- |
| Median duration from IFN-α treatment  to cGVHD, days (range) | 120 (0-1112) |
| Severity of cGVHD, *n* (%) |  |
| Severe | 15 (6.0) |
| Moderate | 59 (23.7) |
| Mild | 55 (22.1) |
| None | 118 (47.5) |
| Number of sites, *n* (%) |  |
| 0 | 118 (47.5) |
| 1 | 69 (27.8) |
| 2 | 33 (13.3) |
| ≥3 | 28 (11.2) |
| Site of cGVHD, *n* (%) |  |
| Skin | 90 (36.3) |
| Mouth | 43 (17.4) |
| Liver | 33 (13.4) |
| Eye | 28 (11.3) |
| Gut | 22 (8.9) |
| Lung | 5 (2.0) |
| Joint | 2 (0.8) |

cGVHD, chronic graft-versus-host disease; IFN-α, interferon-α.

**Supplementary Table 4. Univariable analysis of prognostic factors following preemptive IFN-α therapy**

| **Outcomes** | **HR (95% CI)** | ***P*** | |
| --- | --- | --- | --- |
| **Relapse** |  |  | |
| Sex |  |  | |
| Male | 1 |  | |
| Female | 0.68 (0.38–1.23) | 0.205 | |
| Age |  |  | |
| Adults | 1 |  | |
| Children | 1.56 (0.66–3.67) | 0.309 | |
| Disease risk index before allo-HSCT |  | |  |
| Low risk | 1 |  | |
| Intermediate risk | 2.15 (0.84–5.53) | 0.112 | |
| High risk  First CR induction courses | 4.97 (1.75–14.15) | | 0.003 |
| 1 | 1 | |  |
| >1 | 1.24 (0.67–2.28) | | 0.491 |
| MRD status before allo-HSCT |  | |  |
| Negative | 1 | | 0.182 |
| Positive | 0.62 (0.31–1.25) | |  |
| MRD status before IFN-α therapy |  | |  |
| qPCR/MFC positive alone | 1 | |  |
| qPCR and MFC positive at the same time | 4.72 (2.66–8.38) | | <0.001 |
| Time from allo-HSCT to MRD positive state |  | |  |
| Late-onset MRD | 1 | |  |
| Early-onset MRD | 0.76 (0.40–1.44) | | 0.397 |
| Severity of cGVHD after preemptive IFN-α therapy |  | |  |
| None | 1 | |  |
| Mild to moderate | 0.90 (0.50–1.64) | | 0.738 |
| Severe | 2.06 (0.78–5.45) | | 0.144 |
| Donor type |  | |  |
| Identical sibling donor | 1 | |  |
| Alternative donor | 0.52 (0.29–0.92) | | 0.026 |
| Donor/recipient gender matched |  | |  |
| Female donor/male recipient combination | 1 | |  |
| Others | 1.62 (0.81-3.26) | | 0.174 |
| **NRM** |  | |  |
| Sex |  |  | |
| Male | 1 |  | |
| Female | 0.87 (0.28–2.74) | 0.809 | |
| Age |  |  | |
| Adults | 1 |  | |
| Children | 0.043 (0–334.02) | 0.492 | |
| Disease risk index before allo-HSCT |  |  | |
| Low risk | 1 |  | |
| Intermediate risk | 1.51 (0.33–6.97) | 0.601 | |
| High risk  First CR induction courses | 1.08 (0.10–11.92) | 0.951 | |
| 1 | 1 |  | |
| >1 | 1.31 (0.39–4.35) | 0.662 | |
| MRD status before allo-HSCT |  |  | |
| Negative | 1 |  | |
| Positive | 0.84 (0.18–3.82) | 0.819 | |
| MRD status before IFN-α therapy |  |  | |
| qPCR/MFC positive alone | 1 |  | |
| qPCR and MFC positive at the same time | 2.06 (0.56–7.63) | 0.278 | |
| Time from allo-HSCT to MRD positive state |  |  | |
| Late-onset MRD | 1 |  | |
| Early-onset MRD | 0.39 (0.09–1.80) | 0.229 | |
| Severity of cGVHD after preemptive IFN-α therapy |  |  | |
| None | 1 |  | |
| Mild to moderate | 0.73 (0.02–0.34) | 0.001 | |
| Severe | 0.12 (0.03–0.48) | 0.003 | |
| Donor type |  |  | |
| Identical sibling donor | 1 |  | |
| Alternative donor | 0.25 (0.08–0.78) | 0..018 | |
| Donor/recipient gender matched |  |  | |
| Female donor/male recipient combination | 1 |  | |
| Others | 1.32 (0.29–6.05) |  | |
| **Treatment failure as defined by LFS** |  |  | |
| Sex |  |  | |
| Male | 1 |  | |
| Female | 0.72 (0.42–1.21) | 0.216 | |
| Age |  |  | |
| Adults | 1 |  | |
| Children | 1.21 (0.52–2.81) | 0.657 | |
| Disease risk index before allo-HSCT |  |  | |
| Low risk | 1 |  | |
| Intermediate risk | 1.97 (0.88–4.41) | 0.097 | |
| High risk  First CR induction courses | 3.89 (1.55–9.77) | 0.004 | |
| 1 | 1 |  | |
| >1 | 1.25 (0.73–2.16) | 0.415 | |
| MRD status before allo-HSCT |  |  | |
| Negative | 1 |  | |
| Positive | 0.66 (0.35–1.24) | 0.194 | |
| MRD status before IFN-α therapy |  |  | |
| qPCR/MFC positive alone | 1 |  | |
| qPCR and MFC positive at the same time | 4.07 (2.42–6.83) | <0.001 | |
| Time from allo-HSCT to MRD positive state |  |  | |
| Late-onset MRD | 1 |  | |
| Early-onset MRD | 0.68 (0.38–1.21) | 0.191 | |
| Severity of cGVHD after preemptive IFN-α therapy |  |  | |
| None | 1 |  | |
| Mild to moderate | 0.99 (0.57–1.72) | 0.985 | |
| Severe | 3.33 (1.55–7.14) | 0.002 | |
| Donor type |  |  | |
| Identical sibling donor | 1 |  | |
| Alternative donor | 0.45 (0.27–0.75) | 0.002 | |
| Donor/recipient gender matched |  |  | |
| Female donor/male recipient combination | 1 |  | |
| Others | 1.55 (0.83–2.93) | 0.172 | |
| **Treatment failure as defined by OS** |  |  | |
| Sex |  | |  |
| Male | 1 | |  |
| Female | 0.57 (0.27–1.20) | | 0.138 |
| Age |  | |  |
| Adults | 1 | |  |
| Children | 1.59 (0.56–4.55) | | 0.384 |
| Disease risk index before allo-HSCT |  | |  |
| Low risk | 1 | |  |
| Intermediate risk | 1.30 (0.49–3.46) | | 0.601 |
| High risk  First CR induction courses | 2.93 (0.93–2.56) | | 0.067 |
| 1 | 1 | |  |
| >1 | 1.41 (0.68–2.93) | | 0.355 |
| MRD status before allo-HSCT |  | |  |
| Negative | 1 | |  |
| Positive | 0.44 (0.29–1.71) | | 0.702 |
| MRD status before IFN-α therapy |  | |  |
| qPCR/MFC positive alone | 1 | |  |
| qPCR and MFC positive at the same time | 3.03 (1.46–6.29) | | 0.003 |
| Time from allo-HSCT to MRD positive state |  | |  |
| Late-onset MRD | 1 | |  |
| Early-onset MRD | 0.56 (0.24–1.29) | | 0.173 |
| Severity of cGVHD after preemptive IFN-α therapy |  | |  |
| None | 1 | |  |
| Mild to moderate | 0.73 (0.34–1.60) | | 0.433 |
| Severe | 4.33 (1.67–11.3) | | 0.003 |
| Donor type |  | |  |
| Identical sibling donor | 1 | |  |
| Alternative donor | 0.21 (0.10–0.44) | | <0.001 |
| Donor/recipient gender matched |  | |  |
| Female donor/male recipient combination | 1 | |  |
| Others | 1.62 (0.70–3.75) | | 0.260 |

IFN-α, interferon-a; CI, confidence interval; HR, hazard ratio; allo-HSCT, allogeneic hematopoietic stem cell transplantation; CR, complete remission; qPCR, quantitative polymerase chain reaction; MFC, multiparameter flow cytometry; MRD, measurable residual disease; cGVHD, chronic graft-versus-host disease; NRM, non-relapse mortality; LFS, leukemia-free survival; OS, overall survival.

**Supplementary Figure**

**Supplementary Figure 1.** Cumulative incidence of MRD achieving MRD negative state at 2 years after preemptive IFN-α therapy for patients receiving ISD, HID, and URD/UCB transplantation (A), for patients in low-, intermediate-, and high-risk DRI groups (B), for patients with qPCR/MFC positive alone and both qPCR and MFC positive (C), and for patients without cGVHD, with mild, moderate, and severe cGVHD (D). MRD, measurable residual disease; IFN-α, interferon-α; ISD, identical sibling donor; HID, haploidentical donor; URD, unrelated donor; UCB, unrelated cord blood; qPCR, quantitative polymerase chain reaction; MFC, multiparameter flow cytometry; DRI, disease risk index; cGVHD, chronic graft-versus-host disease;
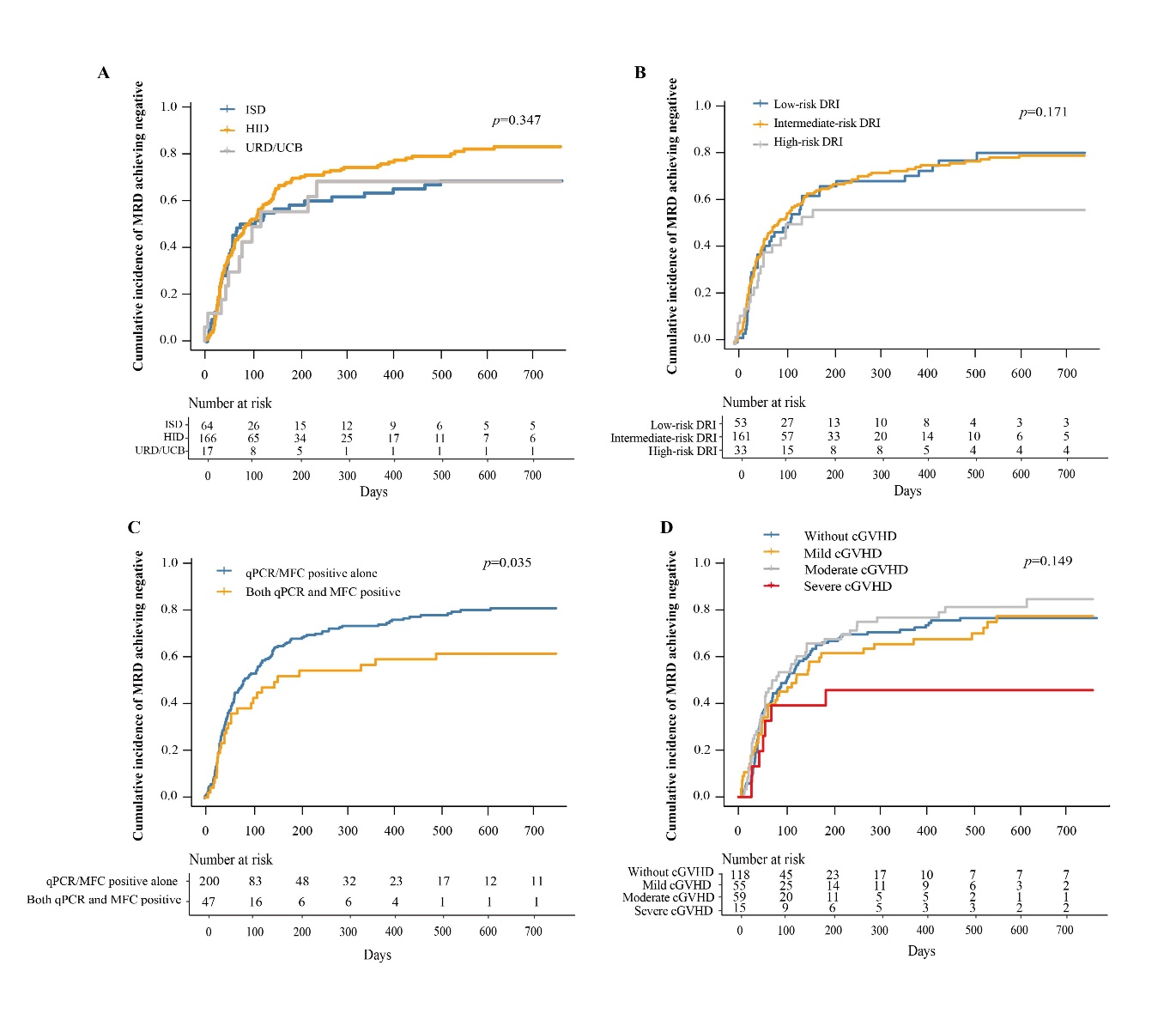


**Supplementary Figure 2**. The cumulative incidence of achieving MRD negative state at 2 years after IFN-α treatment for adults (A) and children (B) without cGVHD, with mild, moderate, or severe cGVHD. MRD, measurable residual disease; IFN-α, interferon-α; cGVHD, chronic graft-versus-host disease;


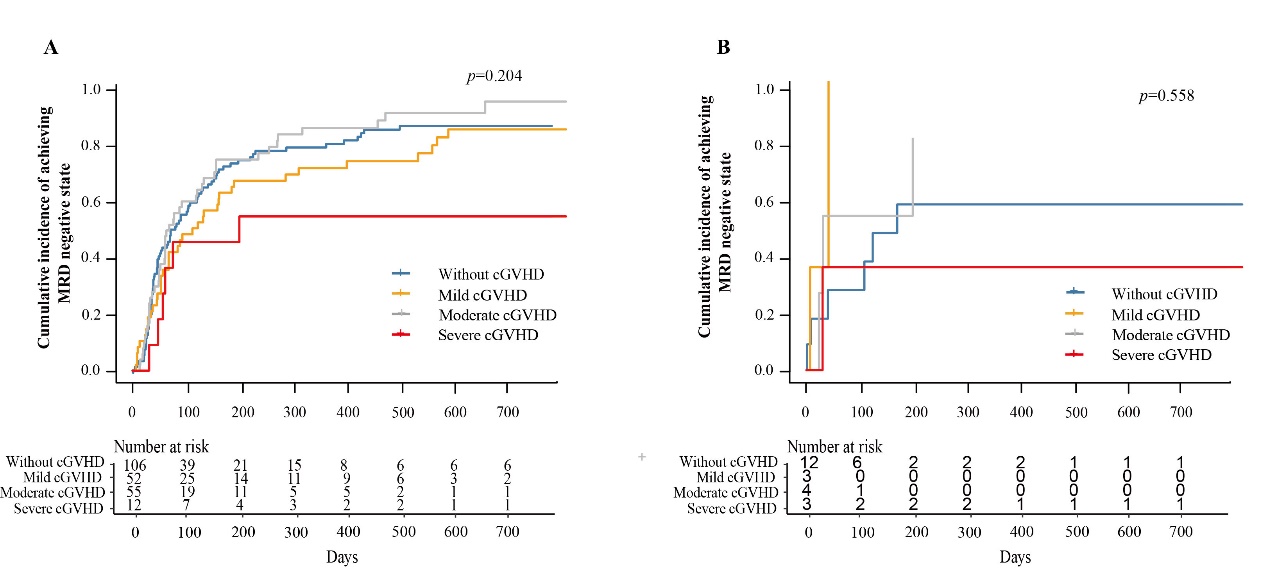


**Supplementary Figure 3.** The cumulative incidence of relapse at 2 years after IFN-α treatment for adults (A) and children (B) without cGVHD, with mild, moderate, or severe cGVHD. cGVHD, chronic graft-versus-host disease;


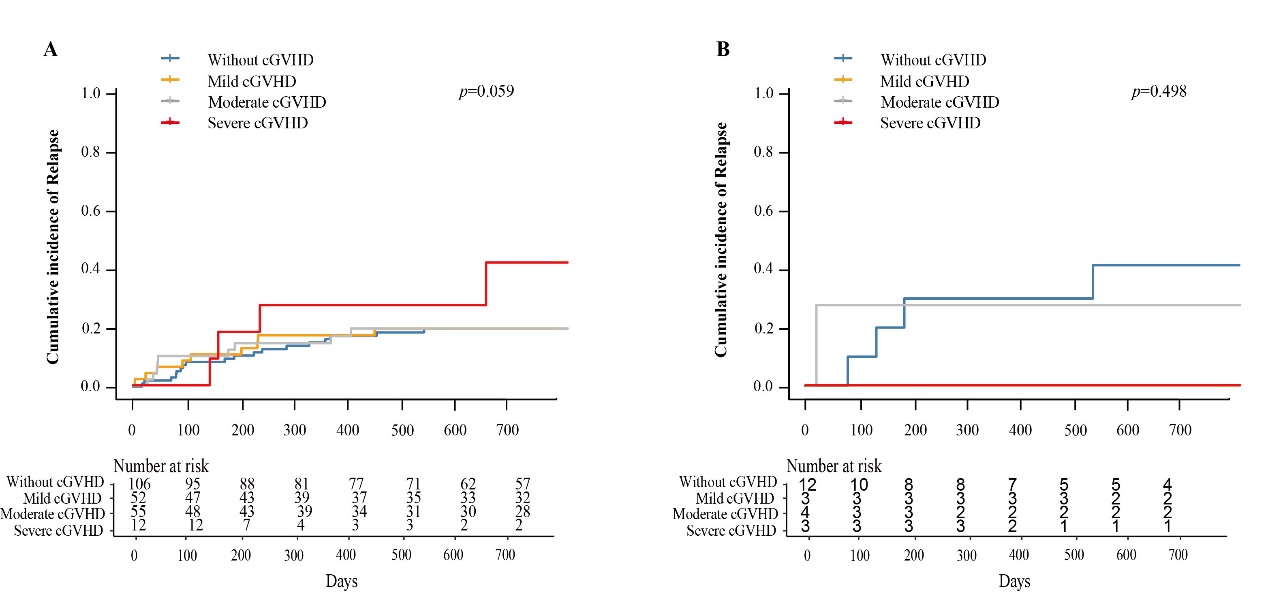


**Supplementary Figure 4.** The cumulative incidence of NRM at 2 years after IFN-α treatment for adults without cGVHD, with mild, moderate, or severe cGVHD. NRM, non-relapse mortality; cGVHD, chronic graft-versus-host disease;


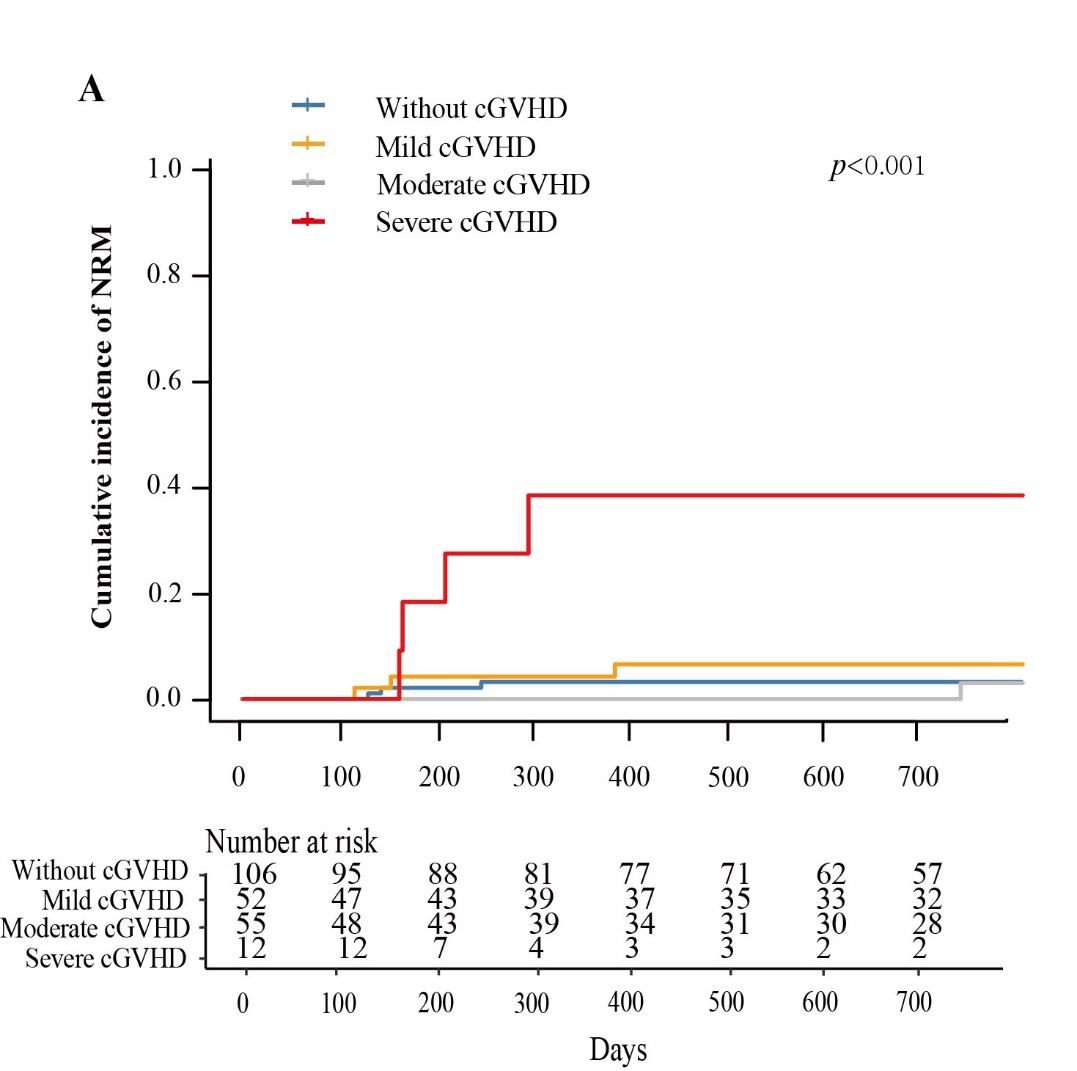


**Supplementary Figure 5.** The probability of LFS at 2 years after IFN-α treatment for adults (A) and children (B) without cGVHD, with mild, moderate, or severe cGVHD. LFS, long-term leukemia-free survival; cGVHD, chronic graft-versus-host disease;


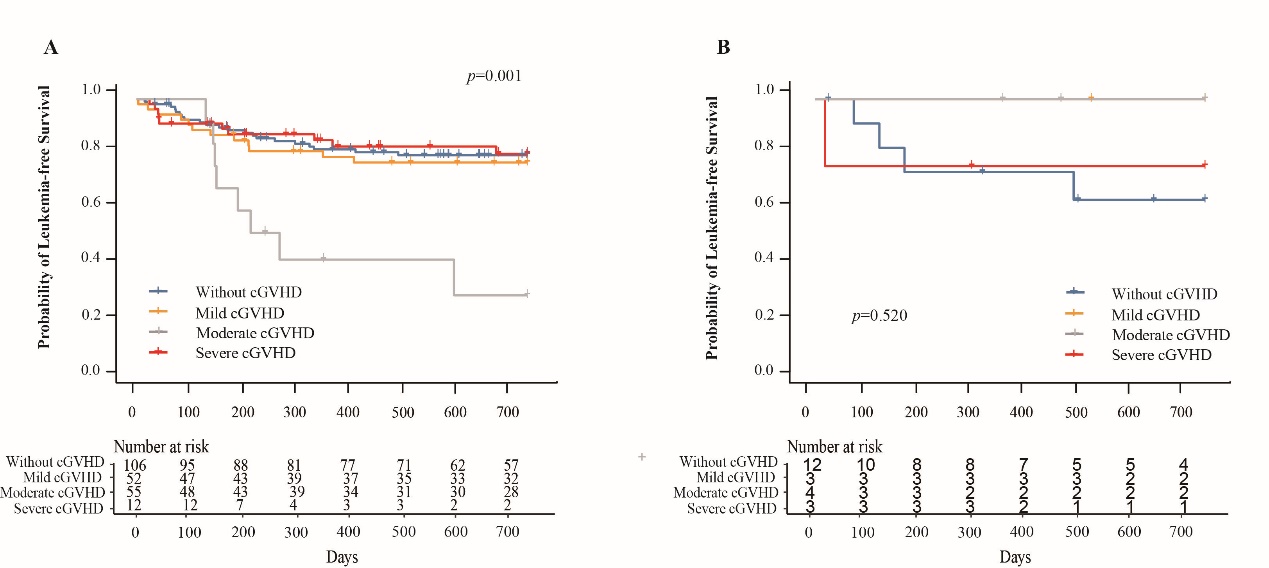


**Supplementary Figure 6.** The probability of OS at 2 years after IFN-α treatment for adults (A) and children (B) without cGVHD, with mild, moderate, or severe cGVHD. OS, overall survival; cGVHD, chronic graft-versus-host disease;


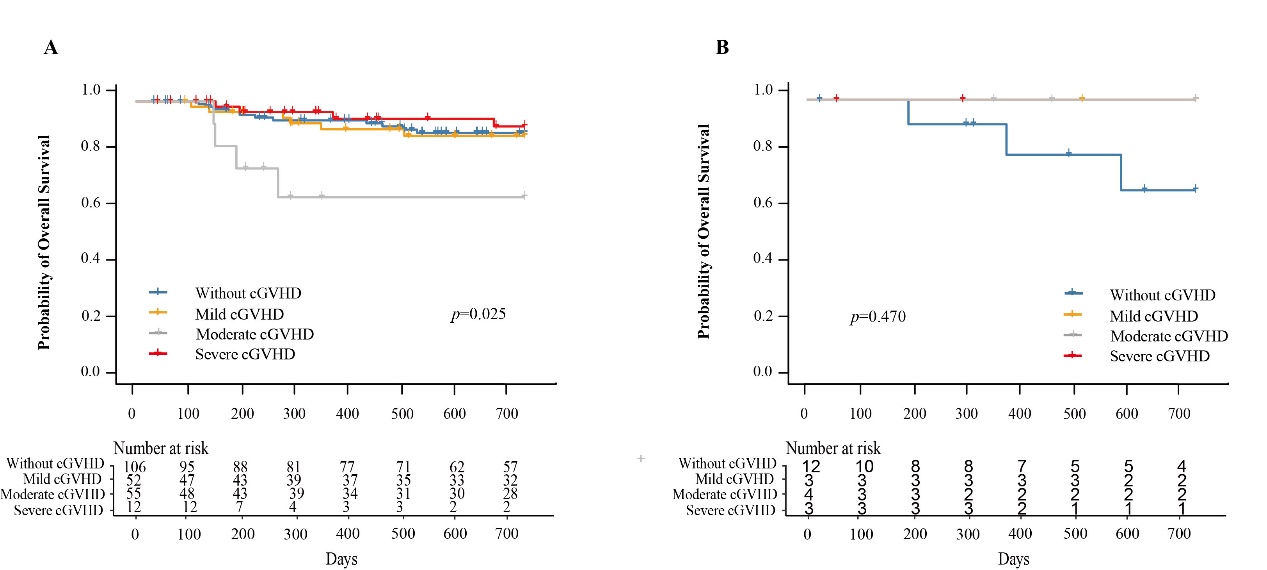


**References**

1. Shen MZ, Zhang XH, Xu LP, et al. Preemptive Interferon-α Therapy Could Protect Against Relapse and Improve Survival of Acute Myeloid Leukemia Patients After Allogeneic Hematopoietic Stem Cell Transplantation: Long-Term Results of Two Registry Studies. Frontiers In Immunology 2022;13:757002.

2. Mo X-D, Tang B-L, Zhang X-H, et al. Comparison of outcomes after umbilical cord blood and unmanipulated haploidentical hematopoietic stem cell transplantation in children with high-risk acute lymphoblastic leukemia. International Journal of Cancer 2016;139:2106-15.

3. Gao XN, Lin J, Wang LJ, et al. Risk factors and clinical outcomes of Epstein-Barr virus DNAemia and post-transplant lymphoproliferative disorders after haploidentical and matched-sibling PBSCT in patients with hematologic malignancies. Annals of Hematology 2019;98:2163-77.

4. Luo Y, Xiao H, Lai X, et al. T-cell-replete haploidentical HSCT with low-dose anti-T-lymphocyte globulin compared with matched sibling HSCT and unrelated HSCT. Blood 2014;124:2735-43.

5. Wang Y, Chen H, Chen J, et al. The consensus on the monitoring, treatment, and prevention of leukemia relapse after allogeneic hematopoietic stem cell transplantation in China. Cancer Letters 2018;438:63-75.
